# Sleep influences the intracerebral EEG pattern of focal cortical dysplasia

**DOI:** 10.1016/j.eplepsyres.2015.03.014

**Published:** 2015-07

**Authors:** Inês Menezes Cordeiro, Nicolas von Ellenrieder, Natalja Zazubovits, François Dubeau, Jean Gotman, Birgit Frauscher

**Affiliations:** a Montreal Neurological Institute and Hospital, McGill University, 3801 University Street, Montreal, Québec H3A 2B4, Canada; b Neurology Department, Centro Hospitalar do Algarve, Faro Hospital, Rua Leao Penedo, 8000-386 Faro, Portugal; c Neurology Department, Medical University of Innsbruck, Anichstrasse 35, 6020 Innsbruck, Austria

**Keywords:** AASM, Polysomnography, Epilepsy, Interictal, Thalamo-cortical, Intracerebral EEG

## Abstract

**Objective:**

Focal cortical dysplasia (FCD) is able to generate an intrinsic pathological EEG activity characterized by a continuous or near-continuous spiking. Different patterns of discharge were described. We examined quantitatively the distribution of the intracerebral FCD patterns in relation to sleep in order to investigate whether this activity is independent of thalamocortical influences.

**Methods:**

We analyzed the first sleep cycle of 5 patients with a diagnosis of FCD type II who underwent combined scalp-intracranial electroencephalography (EEG), and showed an intracranial EEG pattern typical for FCD. Three patterns of FCD intracranial EEG activity were identified in all 5 patients, and visually marked for a maximum of 30 min of each stage (wake, N1, N2, N3, REM): spike or polyspike exceeding 2 Hz (pattern 1), spike or polyspike interrupted by flat periods below 2 Hz (pattern 2) and discharges of >15 Hz low-voltage rhythmic activity with regular morphology (pattern 3). After marking, the percentages of the three patterns across the different stages were calculated.

**Results:**

The three patterns of FCD were present between 45% and 97% of the total time analyzed. Pattern 1 was the predominant pattern in wakefulness (73–100%), N1 (76–97%) and N2 (58–88.5%) in all patients, and in REM in 4 of 5 patients (91–100%). During N2 and N3, there was an increase in pattern 2 in all patients, becoming the predominant pattern in 3 of the 5 patients during N3 (63–89%). Pattern 3 was rare and only sporadically observed during N2 and N3. Wakefulness and REM sleep showed a similar pattern (pattern 1) with a slight amplitude reduction in REM sleep.

**Significance:**

Despite the presence of an almost continuous discharge, sleep is an important modulator of the pathological EEG patterns found in FCD type II. This might suggest that dysplastic tissue is influenced by the thalamo-cortical control mechanisms involved in the generation of sleep.

## Highlights

• We analyze the distribution of intracerebral EEG patterns of FCD in relation to sleep.
• FCD interictal EEG patterns are present between 45% and 97% of the time analyzed.
• Despite almost continuous spiking, sleep is an important modulator of FCD EEG patterns.
• This suggests that dysplastic tissue is under thalamocortical control.

## Introduction

Focal cortical dysplasia (FCD) is a specific malformation of cortical development, often associated with medically refractory epilepsy (Andermann, 2000; Lerner et al., 2009; Semah et al., 1998) and intrinsic epileptogenicity (Battaglia et al., 2013; Chassoux et al., 2000; Palmini et al., 1995).

The intrinsic epileptogenicity of FCD translates into characteristic EEG patterns identified in scalp and intracranial EEG recordings: (1) spike and polyspike and waves with a rhythmic and subcontinuous occurrence, and absence of physiological background; (2) pseudoperiodic spikes or bursts of spikes interrupted by suppression of activity; (3) brief discharges of low-voltage, fast rhythmic activity with regular morphology; and (4) repetitive electrographic seizures with recruiting/derecruiting prolonged trains of rhythmic activity (Chassoux et al., 2000; Francione et al., 2003; Gambardella et al., 1996; Noli et al., 2013; Palmini et al., 1995; Tassi et al., 2002, 2012). Although these patterns are very characteristic for FCD, they were also described in Rasmussen encephalitis and dysembrioplastic neuroepithelial tumors (Chassoux et al., 2000; Palmini et al., 1995).

Sleep is well known to have substantial impact on epileptic discharges showing facilitation of spikes during NREM sleep compared to REM sleep (Malow et al., 1998; Montplaisir et al., 1987; Sammaritano et al., 1991). More specifically, in a very recent study, we showed that epileptic discharges such as interictal spikes and high frequency oscillations are facilitated by widespread high amplitude slow waves occurring during NREM sleep (Frauscher et al., 2015).

Information on the influence of sleep on the pathological EEG activity described in FCD is scarce. FCD type II is associated with higher spike rates during sleep compared to wakefulness in surface EEG (Chassoux et al., 2012) and depth electrode recordings showed an increase of fast discharges during NREM sleep, which tend to spread into contiguous non-lesional areas compared to wakefulness or REM sleep (Francione et al., 2003; Tassi et al., 2002, 2012). In addition, seizures in FCD type II occur predominantly during sleep compared to wakefulness (Chassoux et al., 2012; Noli et al., 2013), and the risk of sleep-related epilepsy (more than 70% of seizures occurring during sleep) (AASM, 2005) is 14 times higher than with other lesions and non-lesional epilepsy independently of the localization of the dysplastic lesion (Nobili et al., 2009).

FCD was suggested to have an intrinsic pacemaker. In order to determine whether epileptogenicity in FCD type II is a truly independent pacemaker or under thalamocortical influence, we investigated systematically and quantitatively the distribution of the various EEG patterns found in patients with FCD type II across the wake sleep cycle.

## Methods

### Selection of patients and nights of recordings

From a total of 57 medically refractory epilepsy patients who underwent presurgical evaluation with combined scalp-intracranial EEG at the Montreal Neurological Institute and Hospital between January 2010 and June 2014, we identified seven patients with a histologically confirmed diagnosis of FCD type II. Five of these seven patients showed the typical FCD EEG patterns during intracerebral EEG recordings. Demographic variables and data on epilepsy history, FCD localization, neuropathological findings, and seizure outcome were collected. The absence of the typical patterns is most likely explained in patient 1 by a sampling problem in a non-lesional FCD IIb (electrode was close, but not inside the lesion); patient 2 had an extensive mesiofrontal FCD IIa with 3 electrodes inside the lesion, but absence of the typical patterns (frequent discharges were present).

We analyzed the first night after 72 h post implantation (to avoid the effect of anesthesia), with no clinical seizures 4 h prior to the nocturnal recording in case of partial or 12 h in case of generalized seizures in order to keep the influence of seizures on the intracranial EEG patterns as low as possible, since there is no literature whether seizures might have an impact on the proportion of the different patterns. We do also not know if electrographic seizures could have an influence, but they generally have no visible impact on the EEG. As they are frequent in some patients, we decided they should not be an exclusion criterion in order not to have to exclude further patients. Data was recorded using the Harmonie EEG system (Stellate, Montreal, Canada).

### Sleep staging and scoring of FCD EEG patterns

**Figure 1:**
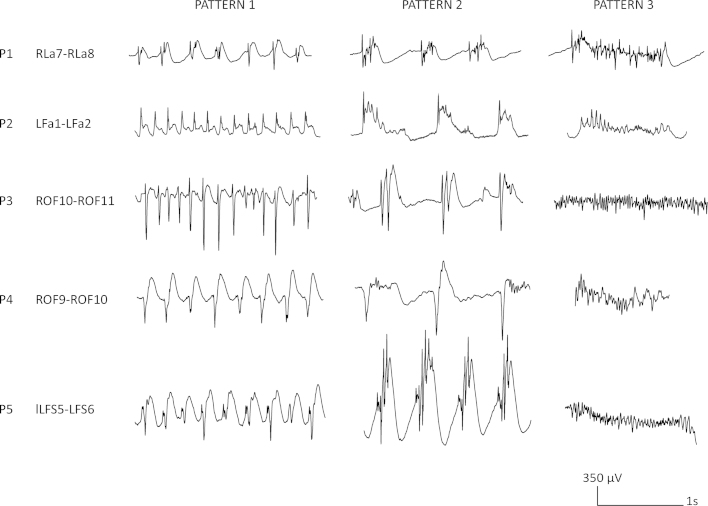
Illustration of the interindividual variation in the morphology of the three patterns observed in FCD. *Pattern 1* was defined as spikes or polyspikes followed or not by a slow wave with a frequency above 2 Hz. *Pattern 2* was defined as spikes or polyspikes followed or not by high amplitude slow waves interrupted by flat periods with a frequency below 2 Hz. *Pattern 3* was defined as discharges of high frequency (>15 Hz), rhythmic activity with regular morphology. Note that for every patient one representative example of each pattern was chosen for the illustration of the typical FCD activity for the respective patients.

The first sleep cycle and its stages (wake, N1, N2, N3, REM) were manually scored according to AASM 2.0 (Berry et al., 2012) in 30 s epochs on the scalp EEG using a bipolar EEG montage (F3-C3, C3-P3, Fz-Cz, Cz-Pz, F4-C4, C4-P4,) by an electrophysiologist specialized in sleep medicine (BF). Following the studies described above (Chassoux et al., 2000; Francione et al., 2003; Gambardella et al., 1996; Noli et al., 2013; Palmini et al., 1995; Tassi et al., 2002, 2012) we defined three interictal EEG patterns: *pattern 1*, spike or polyspike followed or not by a slow wave with a frequency above 2 Hz; *pattern 2*, spike or polyspike followed or not by high amplitude slow waves interrupted by flat periods with a frequency below 2 Hz; and *pattern 3*, discharges of high frequency (>15 Hz), rhythmic activity with regular morphology. Representative examples of the different patterns for each patient are shown in Fig. 1. Of note, only clear-cut spikes, but not rudimentary or abortive spikes were taken into consideration when marking the different patterns.

**Figure 2:**
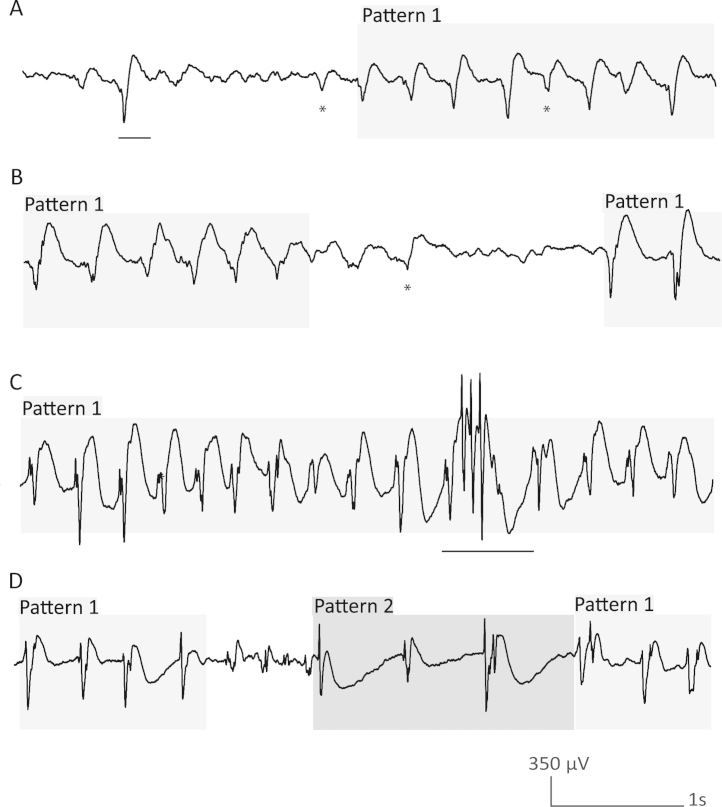
Illustration of the marking of the transitions between the different patterns. (A) The marking of a pattern only starts when the duration is ≥1 s. The first half of the example tracing did not correspond to any of the three patterns. There is only one spike wave complex which was not marked as it is <1 s. The second half corresponds to pattern 1. * marks at most rudimentary but not clear-cut spikes. (B) The marking of a pattern should stop in case of an interruption for more than 1 s. * Note that one element was considered to be a rudimentary but not clear cut spike and was therefore not marked. (C) Interruptions <1 s should not result in stopping of the marking if the same pattern continues after the interruption. In this case one spike wave complex with pattern 2 with a duration <1 s does not stop the marking of pattern 1. (D) The marking of a pattern should stop if another pattern appears for ≥1 s or if after an interruption (even for <1 s) another pattern emerges. In this example, the marking of pattern 1 was stopped due to a <1 s interruption with no pattern (elements were considered to be at most rudimentary but not clear-cut spikes) followed by pattern 2.

These patterns were marked exclusively on intracranial EEG during a maximum of 30 min of each sleep stage during the first sleep cycle. To ensure consistency in the markings, an inter-rater agreement was reached for all patients by two scorers (IMC, BF). Both scorers rated the first 5 min of the respective stages (W, N1, N2, N3, REM) in a common scoring session, and agreed on the presence of pattern 1, 2, or 3 or no pattern. Ambiguous markings were discussed, and a common consensus was reached. The remaining 25 min for each stage were rated by one single scorer (IMC) respecting the consensus reached for the first 5 min. EEG visual analysis was conducted with a time resolution of 30 mm/s at 50 μV/cm using bipolar montages made from adjacent contacts. For each patient, the bipolar channel where the typical epileptiform activity was seen with the highest amplitude was chosen as reference for marking. For wakefulness, the 30 min prior to sleep onset were evaluated. Since seizure activity disrupts the basal epileptiform activity seen in FCD, it was decided that in the case of an electrographic seizure, this activity and the following period was omitted until epileptic activity was back to baseline and a transition into a different sleep stage occurred. A pattern started to be marked when its duration was ≥1 s (Fig. 2A). The marking was stopped when it was interrupted for ≥1 s (Fig. 2B). Interruptions of less than 1 s did not stop the marking if the same pattern continued after the interruption (Fig. 2C), and marking was stopped when a different pattern appeared after the interruption (Fig. 2D).

### Analysis

After visual marking of the patterns in the different stages, the percentage of time of the three patterns and the relative percentages of the different patterns across the various stages were calculated. The absolute percentage of each EEG patterns during wakefulness or a sleep stage was calculated as the time with this pattern divided by the total time of the wake or sleep stage (usually 30 min); example, pattern 1 lasted 20 min of the 30 min of wakefulness; its absolute percentage is 20/30 = 66.7%. The relative percentage of each EEG pattern during wakefulness or a sleep stage was calculated as the time with that pattern divided by the total time in any of the three patterns during the wake or sleep stage; example, during the 30 min of sleep stage N3, pattern 1 occupied 10 min, pattern 2 occupied 13 min and pattern 3 occupied 2 min, for a total of 25 min (during 5 of the 30 min, neither of the 3 patterns was present); the relative percentage of pattern 1 is 10/25 = 40%. The mean values of the relative percentages were used for the graphical illustration of the course of the three patterns across the different sleep stages.

## Results

### Patient characteristics

This analysis comprised five patients (three women) with a diagnosis of histologically confirmed FCD type II (mean age, 27 years and range, 21–38; mean epilepsy duration, 17 years and range, 9–24). FCD lesions were located in the frontal lobe in four patients, and in the parietal lobe in one. The main electroclinical characteristics of the patients are provided in Table 1.

### Percentage of time in FCD pattern

The three EEG patterns of FCD were present almost continuously in two patients throughout the recording (97% and 95% of the time), semicontinuous in one (75%) and present in approximately half of the analysis time in two subjects (45% and 52%). Although the described patterns of FCD could be easily identified in all patients, their morphology varied across individuals (Fig. 1).

### Distribution of the three EEG patterns of FCD across the wake sleep cycle

**Figure 3:**
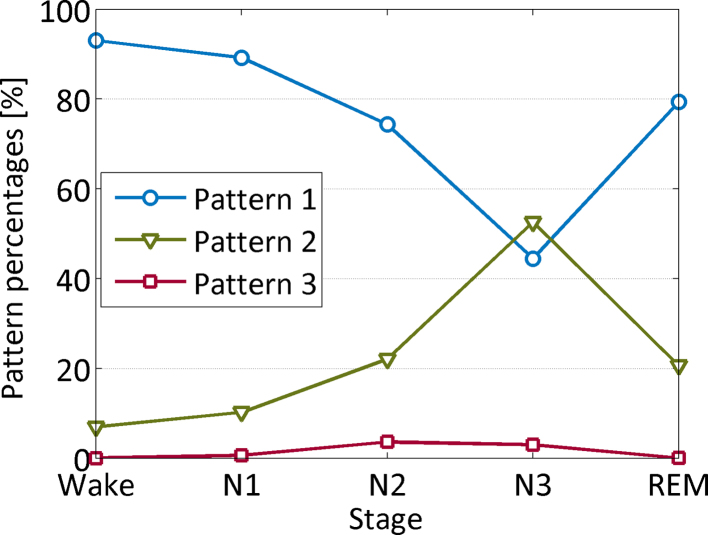
Graph showing the average relative distribution of the three patterns across the different sleep stages. Pattern 1 is the most prominent during wakefulness, N1, N2 and REM sleep, and decreases with sleep depth. In contrast, pattern 2 increases with sleep depth, being the most prominent pattern in N3 sleep. Pattern 3 is rare and predominantly present during NREM sleep stages 2 and 3.

Fig. 3 illustrates the distribution of the three EEG patterns across the wake sleep cycle. Pattern 1 was the predominant pattern in wake, N1 and N2 in all patients and in REM in four of the 5 patients. The percentage of pattern 1 decreased with sleep depth and showed the lowest percentage in N3. REM sleep had a similar percentage of pattern 1 as wakefulness (79 vs. 93%). Pattern 2 was the second most frequent pattern in all patients. In contrast to pattern 1, it increased its percentage with sleep depth in all patients: In three patients, it became the predominant pattern in N3, and in REM sleep, its percentage again decreased in all except one subject. Pattern 3 was the least prevalent pattern, and mainly present in N2 and N3. The relative percentages of the three patterns across wakefulness and sleep stages are given for each patient in Table 2.

### Difference in amplitude between wakefulness and REM sleep

**Figure 4:**
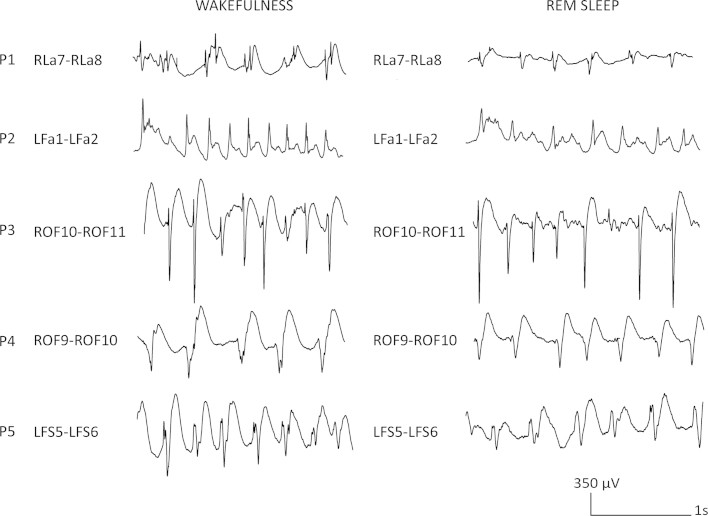
Comparison between examples of representative intracerebral EEG activity in FCD of wakefulness and REM sleep across the five patients. The similarities of the two different stages can be appreciated, albeit amplitude was slightly lower during REM sleep compared to wakefulness.

Wakefulness and REM sleep showed a similar expression with respect to the FCD patterns, with slightly reduced amplitude in REM sleep compared to wakefulness. Fig. 4 illustrates this difference between wakefulness and REM sleep.

## Discussion

Sleep modulates epileptic activity and seizures in patients with FCD. In this study, we aimed to quantify this modulatory effect and investigated the effect of sleep and its respective stages on the intracranial EEG patterns of FCD. Our main finding is that sleep is an important modulator of the frequency of occurrence of the different pathological EEG patterns present in FCD type II.

Sleep modulates the continuous or semicontinous EEG activity generated by FCD during intracerebral EEG recordings. This activity is modulated in a similar way by sleep as the non-continuous interictal epileptic discharges seen in other types of epilepsy (Malow et al., 1998; Montplaisir et al., 1987; Sammaritano et al., 1991). Our findings suggest that dysplastic neurons and circuits are also influenced by thalamo-cortical mechanism involved in the generation of sleep. The distribution of the different intracranial EEG patterns of FCD changed markedly across the different sleep stages: Pattern 1 was predominant during wakefulness, N1, N2 and REM sleep; in contrast, pattern 2 increased with sleep depth and became predominant during N3 sleep; and, finally, pattern 3 although rare, emerged nearly exclusively during NREM sleep stages N2 and N3. This latter finding has been previously described often progressing into seizures (Francione et al., 2003; Tassi et al., 2002, 2012). Since we analyzed only one night without clinical seizures, we do not know if nights with seizures would have higher percentages of pattern 3 than nights without seizures. The activation of pattern 3 by NREM sleep could help to explain why seizures in FCD are more frequent during sleep, irrespective of the localization of the FCD (Chassoux et al., 2012; Nobili et al., 2009; Noli et al., 2013).

Studies showed that in temporal and extratemporal lobe epilepsies, interictal spiking activity propagates to the surrounding non-lesional areas more frequently during NREM sleep (Malow et al., 1998; Montplaisir et al., 1987; Sammaritano et al., 1991). This activation may be due to an increase of cortical neuronal synchronization that occurs during this state, explained by a diminished cholinergic activity and hyperpolarization of the thalamus allowing cortical neurons to fire in a synchronized mode (Brown et al., 2012; Espana and Scammell, 2011). The role of synchronization rather than hyperexcitability in the activation of sleep-related interictal activity was only recently supported by a study showing that epileptic spikes and high frequency oscillations are coupled to the transitions to the “down” or deactivated state, a period of high synchronization (Frauscher et al., 2015). Experimental studies demonstrated, that the inhibitory period following interictal spiking interferes with the generation of seizures, by transiently reducing the excitability of the tissue and hence preventing seizure onset (Engel and Ackermann, 1980; Librizzi and de Curtis, 2003). This finding was confirmed in studies conducted in drug-resistant epileptic patients with FCD and intracerebral electrodes (Curtis et al., 2005; Sato et al., 2014). Pattern 3, in contrast to patterns 1 and 2, shows no period of inhibition, which might explain its facilitating effect and progression into a seizure.

The similarities between wakefulness and REM sleep can be explained by the desynchronized cerebral activity present during both stages. This desynchronization is caused by an increased cholinergic activity responsible for depolarizing the thalamus, which allows transmission of information to the cortex (Brown et al., 2012; Espana and Scammell, 2011). We observed that the amplitude tended to be smaller during REM sleep compared to wakefulness which awaits future quantitative confirmation, but which is in agreement with the general low voltage EEG typical for this state of sleep in the surface EEG (Berry et al., 2012).

Four of the five patients investigated in this study had exclusively or predominantly sleep-related seizures. This finding is in line with the existing literature: In two studies, half of the patients with FCD fulfill the criteria of sleep-related epilepsy and the diagnosis of FCD increases by 14-fold the risk of sleep-related epilepsy independently of the localization of the dysplastic lesion (Chassoux et al., 2012; Nobili et al., 2009) and a more recent study in 31 pediatric epilepsy patients with FCD type II also showed that more than 60% of seizures occurred during sleep (Noli et al., 2013).

The strength of the present study is that we performed a quantitative manual analysis of the different patterns of FCD type II across the different wake and sleep stages. To be as objective as possible, an inter-rater agreement of the first 5 min of each sleep stage was obtained for all patients. It might have been even more reliable if the whole study had been read by two scorers and more than one sleep cycle or night had been studied. One potential limitation resulting from this analysis is that the sample size is comparatively small. Since, however, we obtained similar results across the patients, we think that the results are representative for FCD type II in general.

## Conclusion

This study is the first work to quantitatively assess the distribution of FCD intracerebral EEG patterns in relation to sleep. Despite the presence of an almost continuous discharge, sleep is an important modulator of FCD intracerebral EEG patterns. This might suggest that dysplastic tissue is influenced by the thalamo-cortical control mechanisms involved in the generation of sleep.

Due to the small sample size these findings await confirmation in larger patient samples.

## Conflict of interest

None of the authors has any conflict of interest to disclose.

All authors confirm that they have read the Journal's position on issues involved in ethical publication and affirm that this report is consistent with those guidelines.

## Funding

This work was supported by the Canadian Institutes of Health Research (grant MOP-102710), the Austrian Science Fund (Schrödinger fellowship abroad J3485-B24 to Dr. Birgit Frauscher), and the Austrian Sleep Research Association.

## Figures and Tables

**Table 1:** Demographic and electroclinical information on the investigated patient sample.

ID	Age/gender	Night of sleep study	Seizure distribution	MR Imaging	SEEG implantation sites	SEEG ictal	SEEG interictal	Bipolar channel used for analysis	1 y surgical outcome (Engel class)
1	21/F	6 (7 total)	15/15 NREM (8 clinical, 7 EEG)	R pre-cuneus FCD	R: Ps, Ag, SMg, La,Lp	R: La and Lp > Ag and SMg	R: La, Lp ≫ SMg and Ag	RLa7-RLa8	1
2	30/M	3 (5 total)	25/26 NREM (all clinical)	L anterior cingulate FCD and surgical bed	L:OF, Ca, Cm, SMAa, Fa	L: Fa	L: Fa ≫ OF	LFa1-LFa2	1
3	38/M	6 (6 total)	4/8 wake, 3/8 NREM, 1/8 REM (all clinical)	Normal	R: OF, Ca, SMAa, SMAp, Fp, A, H	R: OF lateral	R: OF lateral	ROF10-ROF11	1
4	26/F	5 (12 total)	4/4 NREM (all clinical)	Normal	R: OF, Ca, Im, Cm, SMAa, SMAp, A, H, Hp	R: OF lateral	R: OF lateral > mid F convexity and ant T neocortex	ROF9-ROF10	1
5	28/F	4 (5 total)	53/87 wake, 34/87 NREM (44 clinical, 43 EEG)*	L F2 FCD	L: OF, Ca, Cm, Fs, H	L: Fs and Cm	L: intermediate and sup. contacts of Fs > Cm	LFs5-LFs6	1

A, amygdala; Ag, angular gyrus; Ca, anterior cingulate gyrus; Cm, middle cingulate gyrus; FCD, focal cortical dysplasia; F, female; Fa, frontal lesion; Fp, frontopolar; Fs, frontal lesion; H, hippocampus; Hp, posterior hippocampus; Im, middle part of the insula; m, male; L, left; La, anterior aspect of the lesion; Lp, posterior aspect of the lesion; OF, orbitofrontal; Ps, superior parietal lobule; R, right; SMAa, anterior part of the supplementary motor area; SMAp, posterior part of the supplementary motor area; SMg, supramarginal gyrus; sup., superficial; y, year.

* Note that the majority of clinical seizures (37/44) occurred during wakefulness.

**Table 2:** Relative percentages of the different EEG patterns of FCD across the sleep stages in the individual patients.

	WAKE and SLEEP STAGES														
	WAKE			N1			N2			N3			REM		
PATTERNS	1	2	3	1	2	3	1	2	3	1	2	3	1	2	3
PATIENTS															
P1	98.1	1.9	0	90.9	9.1	0	72	28.0	0	9.7	89.0	0	90.6	9.4	0
P2	73.4	26.6	0	75.6	24.4	0	68.5	31.4	0.1	34.4	65.6	0	16.2	83.8	0
P3	100.0	0	0	97.3	0	2,7	88.5	1.3	10.3	78.3	12.3	9.4	99.8	0	0.2
P4	99.7	0.3	0	88.4	11.6	0	57.8	42.1	0.1	36.7	63.0	0.3	92.5	7.5	0
P5	94.0	6.0	0	93.8	5.9	0,4	84.9	7.5	7.6	63.2	32.9	4.0	97.7	2.3	0

## References

[bib0005] AASM (2005). International Classification of Sleep Disorders, Revised: Diagnostic and Coding Manual.

[bib0010] Andermann F. (2000). Cortical dysplasias and epilepsy: a review of the architectonic, clinical, and seizure patterns. Adv. Neurol..

[bib0015] Battaglia G., Colciaghi F., Finardi A., Nobili P. (2013). Intrinsic epileptogenicity of dysplastic cortex: converging data from experimental models and human patients. Epilepsia.

[bib0020] Berry R.B., Brook R., Gamaldo C.E., Harding S.M., Marcus C.L., Vaughn B.V. (2012). The AASM Manual for the Scoring of Sleep and Associated Events: Rules, Terminology and Technical Specifications.

[bib0025] Brown R.E., Basheer R., McKenna J.T., Strecker R.E., McCarley R.W. (2012). Control of sleep and wakefulness. Physiol. Rev..

[bib0030] Chassoux F., Devaux B., Landre E., Turak B., Nataf F., Varlet P., Chodkiewicz J.P., Daumas-Duport C. (2000). Stereoelectroencephalography in focal cortical dysplasia: a 3D approach to delineating the dysplastic cortex. Brain.

[bib0035] Chassoux F., Landre E., Mellerio C., Turak B., Mann M.W., Daumas-Duport C., Chiron C., Devaux B. (2012). Type II focal cortical dysplasia: electroclinical phenotype and surgical outcome related to imaging. Epilepsia.

[bib0040] Curtis M., Tassi L., Lo Russo G., Mai R., Cossu M., Francione S. (2005). Increased discharge threshold after an interictal spike in human focal epilepsy. Eur. J. Neurosci..

[bib0045] Engel J., Ackermann R.F. (1980). Interictal EEG spikes correlate with decreased, rather than increased, epileptogenicity in amygdaloid kindled rats. Brain Res..

[bib0050] Espana R.A., Scammell T.E. (2011). Sleep neurobiology from a clinical perspective. Sleep.

[bib0055] Francione S., Nobili L., Cardinale F., Citterio A., Galli C., Tassi L. (2003). Intra-lesional stereo-EEG activity in Taylor's focal cortical dysplasia. Epileptic Disord.

[bib0060] Frauscher B., von Ellenrieder N., Ferrari-Marinho T., Avoli M., Dubeau F., Gotman J. (2015). Facilitation of epileptic activity is mediated by high amplitude slow waves. Brain.

[bib0065] Gambardella A., Palmini A., Andermann F., Dubeau F., Da Costa J.C., Quesney L.F., Andermann E., Olivier A. (1996). Usefulness of focal rhythmic discharges on scalp EEG of patients with focal cortical dysplasia and intractable epilepsy. Electroencephalogr. Clin. Neurophysiol..

[bib0070] Lerner J.T., Salamon N., Hauptman J.S., Velasco T.R., Hemb M., Wu J.Y., Sankar R., Donald Shields W., Engel J., Fried I., Cepeda C., Andre V.M., Levine M.S., Miyata H., Yong W.H., Vinters H.V., Mathern G.W. (2009). Assessment and surgical outcomes for mild type I and severe type II cortical dysplasia: a critical review and the UCLA experience. Epilepsia.

[bib0075] Librizzi L., de Curtis M. (2003). Epileptiform ictal discharges are prevented by periodic interictal spiking in the olfactory cortex. Ann. Neurol..

[bib0080] Malow B.A., Lin X., Kushwaha R., Aldrich M.S. (1998). Interictal spiking increases with sleep depth in temporal lobe epilepsy. Epilepsia.

[bib0085] Montplaisir J., Laverdiere M., Saint-Hilaire J.M., Rouleau I. (1987). Nocturnal sleep recording in partial epilepsy: a study with depth electrodes. J. Clin. Neurophysiol..

[bib0090] Nobili L., Cardinale F., Magliola U., Cicolin A., Didato G., Bramerio M., Fuschillo D., Spreafico R., Mai R., Sartori I., Francione S., Lo Russo G., Castana L., Tassi L., Cossu M. (2009). Taylor's focal cortical dysplasia increases the risk of sleep-related epilepsy. Epilepsia.

[bib0095] Noli D., Bartuluchi M., Gonzalez F.S., Kaltenmeier M.C., Cersosimo R., Rugilo C., Princich J.P., Lubieniecki F., Pomata H., Caraballo R. (2013). Type II focal cortical dysplasia: electroclinical study and surgical outcome in 31 pediatric patients. ChNS.

[bib0100] Palmini A., Gambardella A., Andermann F., Dubeau F., da Costa J.C., Olivier A., Tampieri D., Gloor P., Quesney F., Andermann E. (1995). Intrinsic epileptogenicity of human dysplastic cortex as suggested by corticography and surgical results. Ann. Neurol..

[bib0105] Sammaritano M., Gigli G.L., Gotman J. (1991). Interictal spiking during wakefulness and sleep and the localization of foci in temporal lobe epilepsy. Neurology.

[bib0110] Sato Y., Doesburg S.M., Wong S.M., Boelman C., Ochi A., Otsubo H. (2014). Preictal surrender of post-spike slow waves to spike-related high-frequency oscillations (80–00 Hz) is associated with seizure initiation. Epilepsia.

[bib0115] Semah F., Picot M.C., Adam C., Broglin D., Arzimanoglou A., Bazin B., Cavalcanti D., Baulac M. (1998). Is the underlying cause of epilepsy a major prognostic factor for recurrence?. Neurology.

[bib0120] Tassi L., Colombo N., Garbelli R., Francione S., Lo Russo G., Mai R., Cardinale F., Cossu M., Ferrario A., Galli C., Bramerio M., Citterio A., Spreafico R. (2002). Focal cortical dysplasia: neuropathological subtypes, EEG, neuroimaging and surgical outcome. Brain.

[bib0125] Tassi L., Garbelli R., Colombo N., Bramerio M., Russo G.L., Mai R., Deleo F., Francione S., Nobili L., Spreafico R. (2012). Electroclinical, MRI and surgical outcomes in 100 epileptic patients with type II FCD. Epileptic Disord..

